# LMX1B Activated Circular RNA GFRA1 Modulates the Tumorigenic Properties and Immune Escape of Prostate Cancer

**DOI:** 10.1155/2022/7375879

**Published:** 2022-07-04

**Authors:** Min Meng, Yi-chen Wu

**Affiliations:** Department of Emergency, The Affiliated Huaian No.1 People's Hospital of Nanjing Medical University, Huaian, Jiangsu, China

## Abstract

Prostate cancer (PCa) is the most common cancer affecting men, with increasing global mortality and morbidity rates. Despite the progress in the diagnosis and treatment of PCa, patient outcomes remain poor, and novel therapeutic targets for PCa are urgently needed. Recently, circular RNAs (circRNAs) have been studied in-depth as potential biomarkers for many diseases. In this study, circRNA microarrays using four pairs of PCa tissues were utilized to show that circGFRA1 was upregulated in PCa tumor tissues. CircGFRA1 is suggested to play an oncogene role in PCa progression as the silencing of circGFRA1 inhibited the proliferation, migration, and immune escape activity of PCa cells. Furthermore, by utilizing bioinformatics analysis, RIP, RNA pull-down, and luciferase reporter assays, our results showed that LMX1B could bind to the GFRA1 promoter and regulate circGFRA1 expression in PCa cells and circGFRA1 upregulated HECTD1 expression through sponging miR-3064-5p. This novel LMX1B/circGFRA1/miR-3064-5p/HECTD1 axis identified in PCa provides new insights for developing novel therapeutic strategies for PCa.

## 1. Introduction

Prostate cancer (PCa) is ranked the second major cause of global human malignancy [1], with increasing mortality and morbidity rates in multiple countries in the past two decades [2]. In 2020, PCa contributed to 10% of cancer-related deaths in men [3] and is considerable economic burden for health-care systems. Multiple molecular mechanisms in the initiation or progression of PCa have been explored, including aberrant RNA splicing, irregular ubiquitination and methylation, functional gene dysregulation, and DNA mutations [4, 5], but the specific mechanism underlying PCa initiation and progression remains unclear.

Circular RNAs (circRNAs) are a subtype of noncoding RNAs that are back-spliced and alternative (back-)spliced by a covalently closed loop and have no free terminal ends [6]. Technological innovation of high-through putting sequencing has facilitated the in-depth investigation of circRNAs functions. They can act as decoys for downstream microRNAs (miRNAs) to regulate gene expression and interact with proteins and as scaffolds for circRNA–protein complexes [7]. The functions of circRNAs have been explored in many diseases, such as hepatocellular carcinoma, gastric cancer, renal cell carcinoma, lung cancer, and bladder cancer ([8–13]). Furthermore, accumulating evidence suggests the essential role of circRNA in PCa progression. It has been shown that circ_0057558 modulates the proliferation of PCa cells via the miR-206/USP33/c-Myc pathway [14]. NF-*κ*B upregulated circNOLC1 regulates PCa progression through the miR-647/PAQR4 axis [15], circSMARCA5 facilitates PCa cellular behaviors through the miR-181b-5p/miR-17-3p-TIMP3 axis [16], and circGNG4 promoted PCa progression through modulating the miR-223/EYA3/c-myc pathway [17].

This study aimed to identify a novel circRNA utilizing circRNA microarray analysis using four pairs of PCa tumor tissues and normal adjacent tissues. The analysis revealed that circGFRA1 was markedly upregulated in PCa tumor tissues and is induced by LMX1B, playing a promotive effect in PCa progression via the miR-3064-5p/HECTD1 axis.

## 2. Materials and Methods

### 2.1. Human Samples

Four pairs of PCa tumor tissues and adjacent normal tissues were obtained from the Affiliated Huaian No.1 People's Hospital of Nanjing Medical University from 2019 to 2021. All patients had not undergone chemotherapy or radiotherapy before surgical resection and provided informed content. All tissues were verified by two pathologists, and the study protocol was approved by the Ethics Committee of the Affiliated Huaian No.1 People's Hospital of Nanjing Medical University.

### 2.2. Cell Culture and Treatment

Prostate cancer cell lines (DU145, PC-3, LNCap, and 22Rv1) and normal prostatic epithelial cells (RWPE-1) were purchased from the American Type Culture Collection and cultured in DMEM medium (Invitrogen, Thermo Fisher, USA) supplemented with 10% fetal bovine serum (Gibco) and 100 units/ml penicillin and 100 g/ml streptomycin in a 37°C environment with 5% CO_2_. The siRNA targets circGFRA1, LMX1B, and HECTD1, pcDNA3.1-LMX1B vector, and miR-3064-5p mimic were synthesized and obtained from Genepharm (Shanghai, China). All transfections were performed using a Lipofectamine® 3000 kit (Invitrogen; Thermo Fisher, USA) following the manufacturer's instructions.

### 2.3. RNA Real-Time qPCR (RT-qPCR)

Total RNA extraction was conducted using TRIzol® (Invitrogen; Thermo Fisher, USA), and cDNA was synthesized using a miScript II RT kit (Qiagen, Haidian, Beijing, China). RT-qPCR experiment was performed using a miScript SYBR Green PCR kit (Qiagen, Haidian, Beijing, China) on an ABI 7600 cycler (Applied Biosystems), with GAPDH and U6 used as internal controls. The relative expression was quantified by the 2^-*ΔΔ*Ct^ method. The primers used are as follows:

circGFRA1; F: CCTCCGGGTTAAGAACAAGC, R: CTGGCTGGCAGTTGGTAAAA. GFRA1; F: TGTCAGCAGCTGTCTAAAGG, R: CTTCTGTGCCTGTAAATTTGCA. miR-3064-5p; F: ATCGTCTGGCTGTTGTGGT, R: GTGCAGGGTCCGAGGT. HECTD1; F: AATGAACCGGG TCAACTGC, R: TGTGTTTGTCCACTGGCATT. GAPDH; F: ATGGGGAAGGTGAAGGTCG, R: GGGGTCATTGATGGCAACAATA. U6; F: CTCGCTTCGGCACA, R: AACGCTTCACGAATTTGCGT.

### 2.4. Western Blotting

RIPA lysis buffer (Beyotime, Shanghai, China) was used to extract proteins from cells and tissues. The protein samples were separated on a 10% SDS-PAGE gel and transferred to PVDF membranes (Millipore). The membranes were blocked with 5% nonfat milk before incubation with primary antibodies (LMX1B: CST, 1 : 1000, 13457S; HECTD1: Abcam, 1 : 5000, ab101992, GAPDH: Abcam, 1 *μ*g/ml, ab37168) overnight at 4°C and then incubated with secondary antibodies for 2 h at room temperature. An enhanced chemiluminescent (ECL) system (Beyotime) was used to visualize the protein bands with GAPDH used as the internal control.

### 2.5. Cell Proliferation Detection

The Cell Counting Kit-8 (CCK-8) and 5-ethynyl-2′-deoxyuridine (EdU) assay were used to detect cell proliferation levels. For the CCK-8 assay, cells were seeded in a 96-well plate and cultured at 37°C with 5% CO_2_ for three days before the addition of 10 *μ*l of CCK-8 solution at each time point and cultured for 4 h. The optical density was measured at 450 nm. For the EdU assay, the cells were incubated in DMEM with 10 mM EdU for one day. Subsequently, cells were fixed with 4% paraformaldehyde for 30 min and neutralized with 2 mg/ml glycine for 10 min before washing with PBST. The cells were then stained with Apollo and Hoechst33342.

### 2.6. Cell Migration

After transfection, the cells were washed in PBS, resuspended in serum-free medium (200 *μ*l), and placed in the upper chamber for migration detection. The chamber was supplemented with 600 *μ*l of complete medium containing 10% serum. After 24 h, the cells were fixed formaldehyde and stained with crystal violet (Sigma-Aldrich; Merck KGaA) before visualization using an Olympus light microscope.

### 2.7. Bioinformatics Analysis

The National Center for Biotechnology Information (NCBI; https://www.ncbi.nlm.nih.gov/), the University of California Santa Cruz (UCSC; http://genome.ucsc.edu/), and the JASPAR database (https://jaspar.genereg.net/) were used to investigate the upstream factor of circGFRA1. The miRNA target of circGFRA1 was predicted by the ENCORI database (https://starbase.sysu.edu.cn/index.php) under CLIP data, high stringency (≥3), and class (8mer) condition. The mRNA target of miR-3064-5p was analyzed by utilizing microT (http://diana.imis.athena-innovation.gr/), miRmap (https://mirmap.ezlab.org/), and PicTar (https://pictar.mdc-berlin.de/) databases under CLIP data, strict stringency (≥5), degradome data, and medium stringency (≥2).

### 2.8. Enzyme-Linked Immunosorbent Assay (ELISA)

The supernatants were collected from treated cells to quantify the production of immunosuppressive factors vascular endothelial growth factor (VEGF), IL-10, and transforming growth factor-*β*1 (TGF-*β*1) using commercially available ELISAs (Beckman Coulter Life Sciences, Brea, CA, USA).

### 2.9. Cytotoxicity Activity Analysis

To validate the cytokine-induced killer (CIK) cell-induced cytotoxicity towards PCa cells, the cells were co-cultured at 10: 1, 20 : 1, and 40 : 1 ratios, respectively, in a 37°C environment with 5% CO_2_ for one day. Subsequently, 20 *μ*l of CCK-8 solution was added to 100 *μ*l of cell medium for 4 h before the optical density was measured at 450 nm. The survival rate was calculated as follows: survival (%) = (functional&target cell mixture − functional cell)/target cell∗100%.

### 2.10. RNA-Binding Protein Immunoprecipitation (RIP)

Cell lysates were incubated with sepharose beads (Bio-Rad, USA) prefixed with argonaute-2 (Ago2) or immunoglobulin G (IgG). The beads were washed and analyzed by qRT-PCR.

### 2.11. RNA Pull-down

CircGFRA1 or miR-3064-5p was biotinylated to construct bio-circGFRA1 or bio-miR-3064-5p probes. Subsequently, 2 *μ*g cell lysate was incubated with 100 pmol biotinylated probes before the addition of streptavidin agarose beads for 1 h at room temperature. The beads were boiled in SDS and analyzed by RT-qPCR.

### 2.12. Dual-Luciferase Reporter Gene Assay

Wild-type (WT) or mutant (Mut) sequences of the target gene were inserted into psiCHECK2 plasmids. (Thermo Fisher, USA). PCa cells were cultured in a 24-well plate for 24 h (2 × 10^4^ cells per well) and then co-transfected with WT or Mut reporter plasmids (containing circGFRA1 or HECTD1 3′-UTR sequence) or miR-3064-5p mimics to verify the relationship among circGFRA1/miR-3064-5p/HECTD1 axis, and cells were co-transfected with WT or Mut reporter plasmids (containing circGFRA1 or HECTD1 3′-UTR sequence) and miR-3064-5p mimics. The association between LMX1B and GFRA1 promoter was examined through co-transfecting WT or Mut reporter plasmids (region 2 or 4 sequences of GFRA1 promoter) and LMX1B vectors into cells. Transfections were performed using Lipofectamine® 3000 (Invitrogen, USA), and luminescence was detected 48 h later using the dual-luciferase detection kit (Promega, USA).

### 2.13. Statistical Analysis

The statistical analyses were performed using SPSS 19.0 software (IBM Corporation, USA). The data were presented as mean ± SD and subjected to one-way ANOVA and Student *t*-tests. All experiments were performed in triplicate, and a *P* value <0.05 was considered statistically different.

## 3. Results

### 3.1. Expression of circGFRA1 in PCa

**Figure 1:**
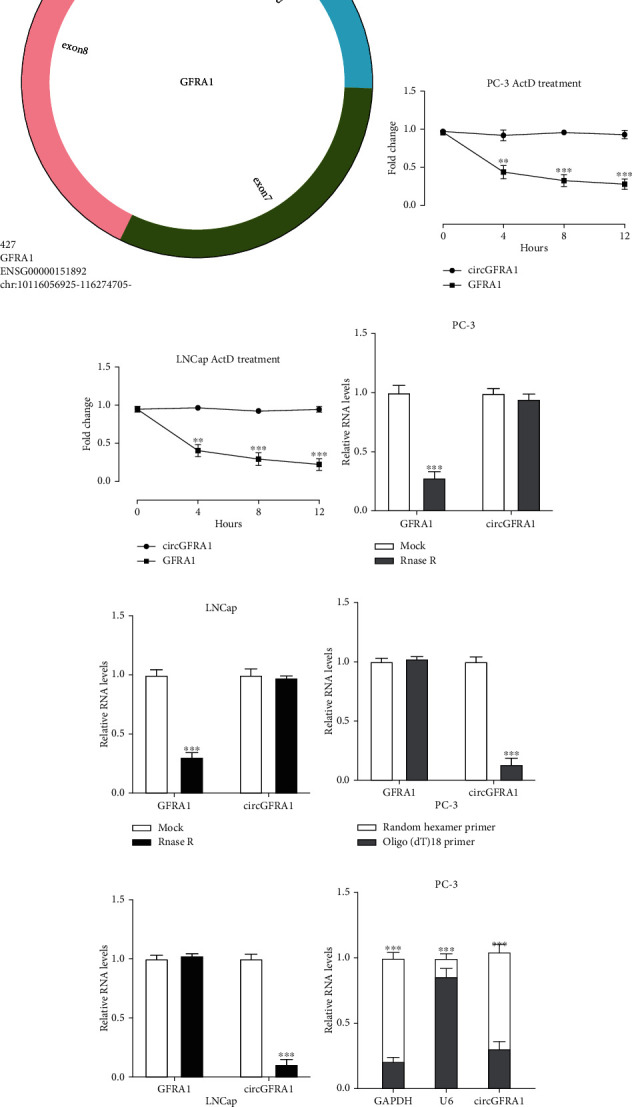
Expression patterns of circGFRA1 in PCa. (a) Microarray analysis of four pairs of prostate cancer tissues. (b) Relative expression of circGFRA1 in PCa cell lines showing circGFRA1 expression was significantly upregulated in PCa cell lines compared to RWPE-1 cells (∗∗*P* < 0.01). (c) Schematic illustration of circGFRA1. (d and e) The effect of ActD treatment on the stability of circGFRA1 and linear form GFRA1 in PC-3 and LNCap cells. (f and g) The effect of RNase R on the expression of circGFRA1 and linear form GFRA1 in PC-3 and LNCap. (h and i) Expression of circGFRA1 in PC-3 and LNCap cells analyzed by qRT-PCR after normalization with random primers and oligo (dT) 18 primers. (j and k) The distribution of circGFRA1 in PC-3 and LNCap cells was detected by cellular fragment assay. Data are presented as mean ± standard error. ∗∗*P* < 0.01, ∗∗∗*P* < 0.001.

The expression profile of circRNAs in four pairs of PCa tissues was examined by microarray analysis (Figure 1(a)), showing that circGFRA1 (hsa_circ_005239, chr10:116,059,925–116,274,705), which is derived from gene GFRA1 (GDNF family receptor alpha 1), was abundantly expressed in all four PCa tumor tissues compared to normal tissues. CircGFRA1 was also upregulated in PCa cell lines, especially in PC-3 and LNCap cells (Figure 1(b)). The schematic diagram of circGFRA1 is shown in Figure 1(c), and to confirm that circGFRA1 was indeed circular in PCa cells, cells are treated with the transcription inhibitor actinomycin D (ActD) showing that the half-life of circGFRA1 was significantly longer than GFRA1 mRNA (Figures 1(d) and 1(e)). Moreover, circGFRA1 was more resistant to RNase R digestion than its linear form (Figures 1(f) and 1(g)). Next, the use of random and oligo(dT)18 primers in reverse transcription to deplete circRNAs in the 3′ pA tail, as expected, reduced circGFRA1 level in PC-3 and LNCap cells compared to the linear form (Figures 1(h) and 1(i)). In addition, the subcellular location assay showed that circGFRA1 was mainly distributed in the cell cytoplasm (Figures 1(j) and 1(k)), indicating that circGFRA1 might participate in the cellular behaviors of PCa.

### 3.2. Silencing circGFRA1 Attenuates the Tumorigenic Properties and Immune Escape of PCa

**Figure 2:**
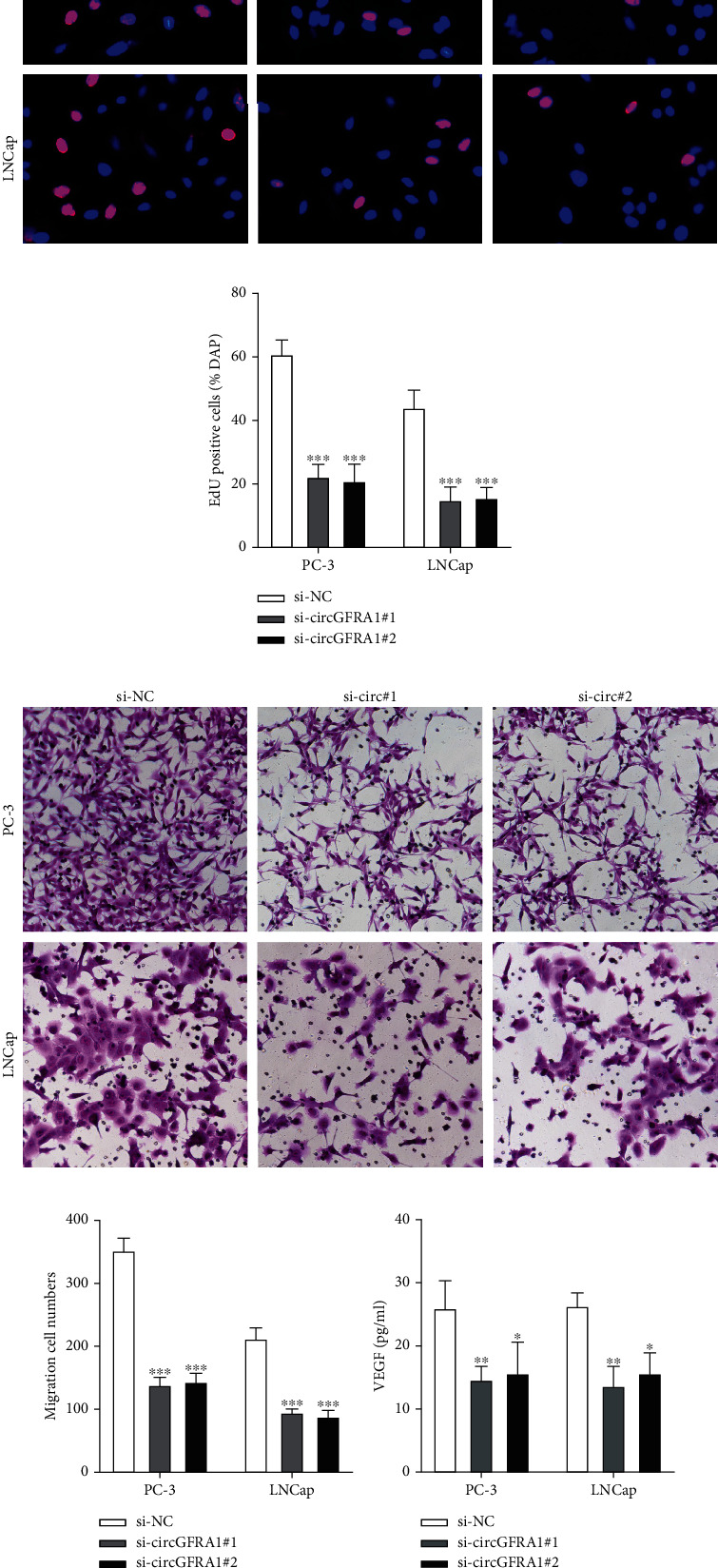
Silencing circGFRA1 attenuates the tumorigenic properties and immune escape of PCa. (a and b) Loss-of-function cell models were constructed via transfecting si-NC, si-circGRFA1#1, and si- circGRFA1#2 into PC-3 and LNCap cells. CircGRFA1 and GFRA1 expression were measured by qRT-PCR. (c and d) Cell proliferation was detected by CCK-8. (e and f) EdU assay was performed to evaluate cell proliferation. (g and h) Cell migration was assessed by the transwell assay. (i) VEGF content of PCa cell supernatants. (j) IL-10 content of PCa cell supernatants. (k) TGF-*β*1 content of PCa cell supernatants. (l and m) The cytotoxicity of CIK cells to PC-3 and LNCap cells was determined via CCK-8 assay. Results from the si-NC group were used as a control. Data are presented as mean ± standard error. ∗*P* < 0.05, ∗∗*P* < 0.01, ∗∗∗*P* < 0.001.

To study the function of circGFRA1, circGFRA1 but not GFRA1 was silenced in PC-3 and LNCap cells (Figures 2(a) and 2(b)), resulting in reduced cell proliferation (Figures 2(c)–2(f)) and cell migration (Figures 2(g) and 2(h)). It has been demonstrated that immune escape is essential for the development of PCa [18], and we determined the expression of immunosuppressive factors VEGF, IL-10, and TGF-*β*1 in the supernatant of circGFRA1 silenced PC-3 and LNCap cells. As shown in Figures 2(i)–2(k), VEGF, IL-10, and TGF-*β*1 are markedly decreased, and CIK cell-induced cytotoxic activity against circGFRA1 silenced cells was higher compared to normal control cells in the same conditions (Figures 2(l) and 2(m)), suggesting that silencing circGFRA1 inhibits the immune escape of PCa.

### 3.3. LMX1B Binds to GFRA1 and Upregulates circGFRA1 Expression in PCa Cells

**Figure 3:**
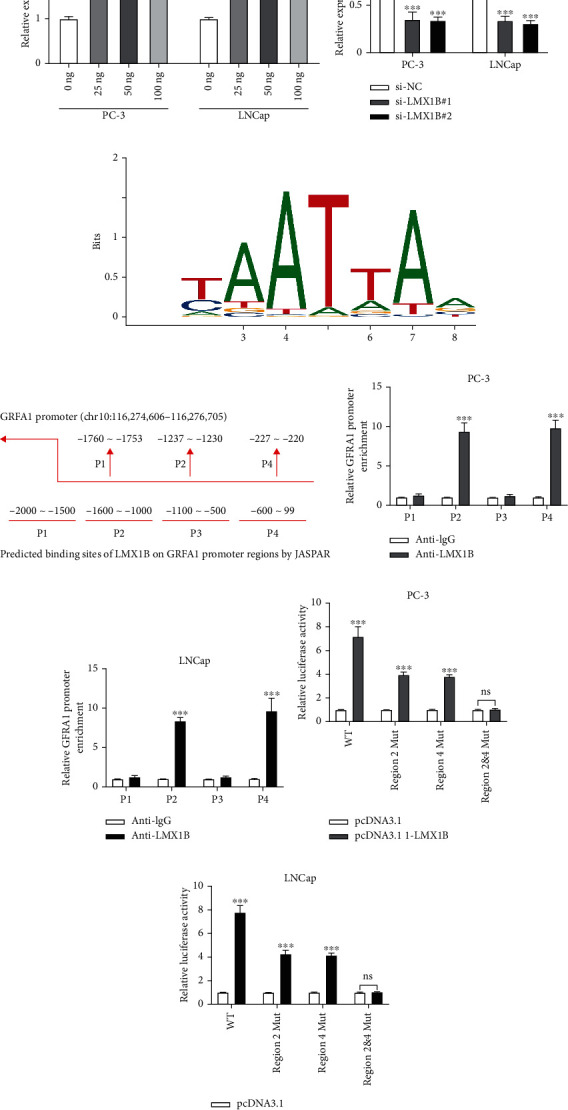
LMX1B binds to circGFRA1 and upregulates circGFRA1 expression in PCa cells. (a) Relative circGFRA1 expression in PC-3 and LNCap cells upon 0/20/50/100 ng LMX1B transfection and circGFRA1 expression upon 0 ng LMX1B transfection were used as control. (b) Relative circGFRA1 expression in PCa cells upon si-NC, si-LMX1B#1, and si-LMX1B#2 transfection results from si-NC group was used as control. (c) The predicted binding motif of LMX1B obtained from JASPAR. (d) The predicted binding motif of GFRA1 promoter regions obtained from JASPAR. (e and f) ChIP analysis of enrichment of LMX1B on the GFRA1 promoter in PC-3 and LNCap cells. IgG was used as a negative control. (g and h) Quantification of the luciferase activity of the wild-type or mutant GFRA1 promoter reporter in PC-3 and LNCap cells. Data are presented as mean ± standard error. ∗*P* < 0.05, ∗∗∗*P* < 0.001.

Interestingly, putative LMX1B binding sites were identified in promoter regions of GFRA1, so the effects of LMX1B on circGFRA1 expression in PC-3 and LNCap cells were quantified by qRT-PCR, showing that circGFRA1 expression was upregulated by LMX1B overexpression in a dose-dependent manner (Figure 3(a)) and downregulated by LMX1B knockdown (Figure 3(b)). The schematic diagram of the putative binding sites of LMX1B (Figure 3(c)) or GFRA1 promoter regions is shown in Figure 3(d). The ChIP assay revealed that the P2 and P4 regions on the GFRA1 promoter were markedly enriched by anti-LMX1B compared to anti-IgG in PC-3 and LNCap cells (Figures 3(e) and 3(f)). Furthermore, the luciferase activity increased when the P2 or P4 regions on the GFRA1 promoter were mutated (Figure 3(g)) and was unchanged when both P2 and P4 regions on the GFRA1 promoter were mutated (Figure 3(h)). Taken together, these results suggest that LMX1B regulates circGFRA1 expression in PCa cells.

### 3.4. LMX1B Modulates the Tumorigenic Properties and Immune Escape of PCa

**Figure 4:**
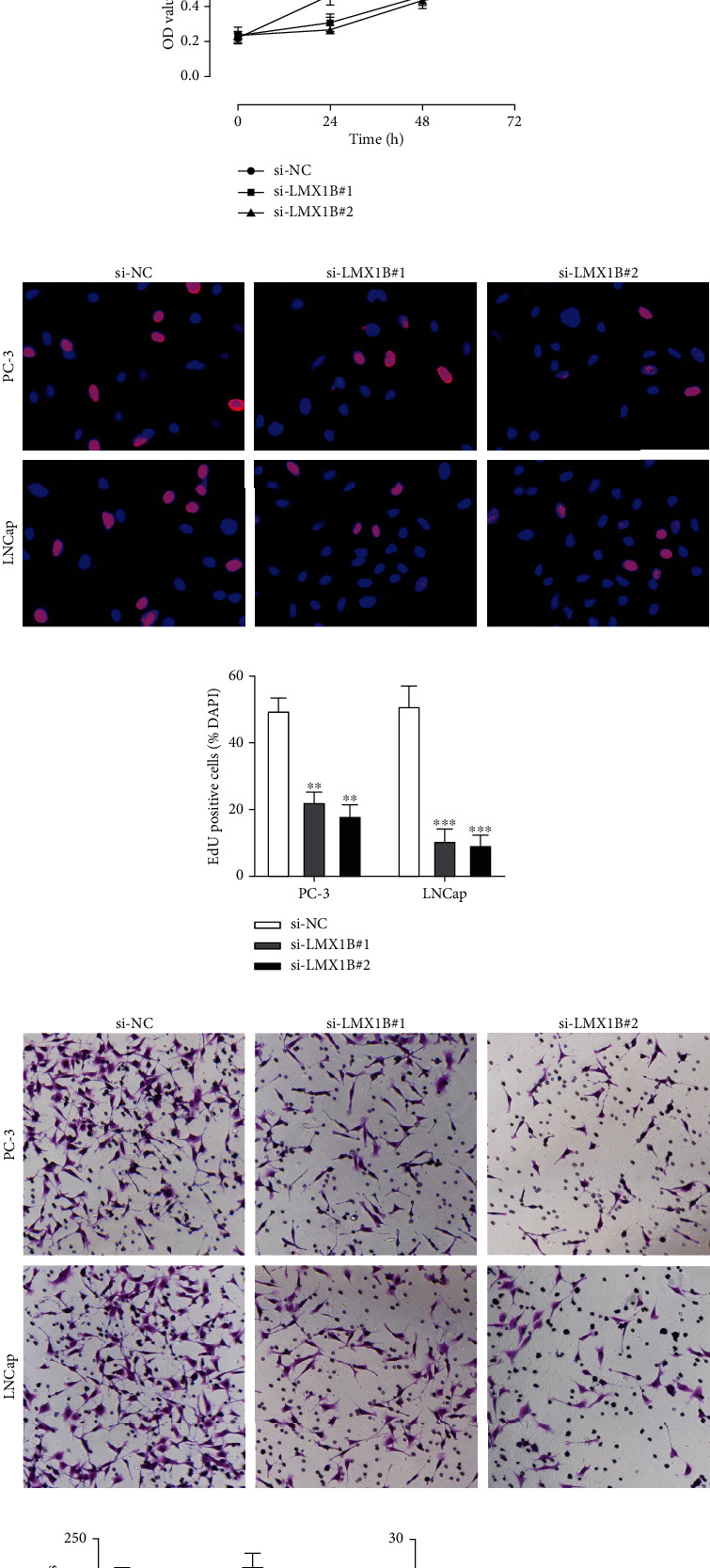
LMX1B is upregulated in PCa and modulates the tumorigenic properties and immune escape of PCa. (a) Relative expression of LMX1B in thirty pairs of PCa tumor tissues and comparative normal tissues. (b) Protein expression of LMX1B in randomly selected five pairs of tumor tissues and comparative normal tissues. (c) Relative expression of LMX1B in PCa cell lines (RWPE-1, DU145, PC-3, LNCap, and 22Rv1 cell lines) with expression in RWPE-1 cells used as the control. (d and e) Loss-of-function cell models were constructed via transfecting si-NC, si-LMX1B#1, and si-LMX1B#2 into PC-3 and LNCap cells, respectively. The mRNA and protein expression of LMX1B were measured by qRT-PCR and western blot. (f and g) Cell proliferation was detected by CCK-8. (h and i) EdU assay to evaluate cell proliferation. (j and k) Cell migration was assessed by the transwell assay. (l) VEGF content of PCa cell supernatants. (m) IL-10 content of PCa cell supernatants. (n) TGF-*β*1 content of PCa cell supernatants. (o and p) The cytotoxicity of CIK cells to PC-3 and LNCap cells was determined via CCK-8 assay with results from the si-NC group as control. Data are presented as mean ± standard error. ∗*P* < 0.05, ∗∗*P* < 0.01, ∗∗∗*P* < 0.001.

To demonstrate the role of LMX1B in PCa, LMX1B expression was analyzed in thirty pairs of PCa tissues by qRT-PCR and then validated in five pairs of PCa tissues by western blotting. As shown in Figures 4(a) and 4(b), the expression of LMX1B in PCa tumors is markedly higher than in normal tissues. Furthermore, LMX1B was upregulated in PCa cell lines (Figure 4(c)). Loss-of-function studies by stably knocking down LMX1B expression in PC-3 and LNCap cells were conducted (Figures 4(d) and 4(e)) showing that LMX1B knockdown significantly inhibited cell proliferation (Figures 4(f) and 4(i)) and markedly suppressed cell migration level (Figures 4(j) and 4(k)). Moreover, LMX1B knockdown significantly decreased VEGF, IL-10, and TGF-*β*1 in the supernatant of PCa cells (Figures 4(l)–4(n)) and markedly increased the CIK cell-induced cytotoxic activity towards PCa cells (Figures 4(l) and 4(m)).

### 3.5. CircGFRA1 Functions as a Molecular Sponge for miR-3064-5p

**Figure 5:**
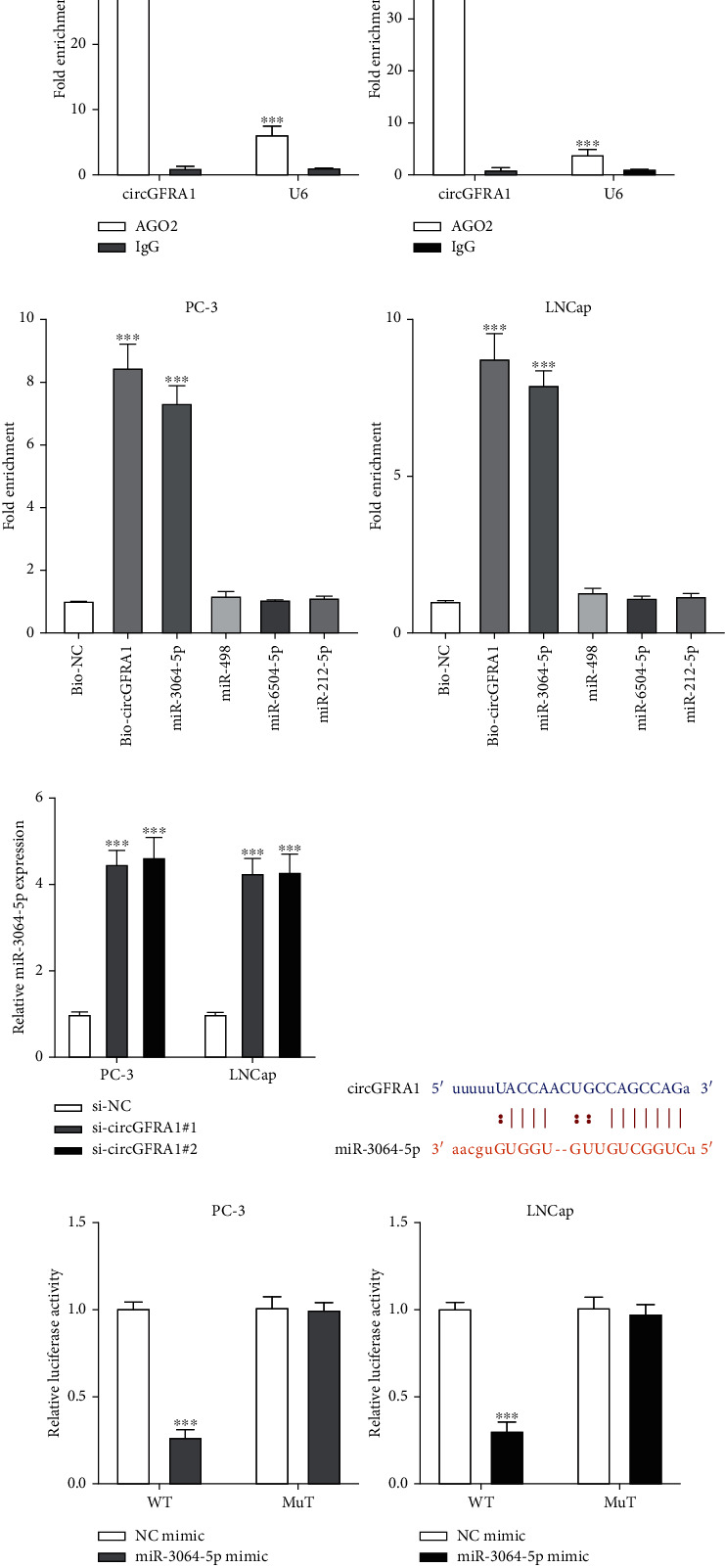
CircGFRA1 sponges miR-3064-5p. (a) The enrichment of circGFRA1 in the RISC of PC-3 and LNCap cells using anti-Ago2 or IgG antibody with the IgG group results used as a negative control. (b) Bio-RNA pull-down assay to evaluate the relative enrichment of putative miRNA targets of circGFRA1 in probes in PC-3 and LNCap cells, and bio-NC group was used as control. (c) Relative miR-3064-3p expression in circGFRA1 knockdown cells was determined by qRT-PCR using the si-NC group as control. (d) Schematic of circGFRA1 illustrating the position of the miR-3064-5p binding site. (e) Relative luciferase activity in PC-3 and LNCap cells co-transfected with circGFRA1-WT or circGFRA1-MUT and miR-NC mimic and miR-3064-5p mimic using the NC mimic group results as control. Data are presented as mean ± standard error. ∗*P* < 0.05, ∗∗∗*P* < 0.001.

Since circRNA can function as a molecular sponge for microRNAs (miRNA), we analyzed whether circGFRA1 binds to miRNA through a competitive endogenous RNA (ceRNA) mechanism. It is well known that AGO2 is essential for the biogenesis and mature of miRNAs, and our results showed that circGFRA1 can bind to AGO2 suggesting that circGFRA1 may bind to miRNA in PCa cells (Figure 5(a)). Subsequent bioinformatics analysis identified four miRNAs with relatively high scores, and the qRT-PCR assay results showed that only miR-3064-5p was abundantly pulled down by the circGFRA1 probe in PC-3 and LNCap cells (Figure 5(b)), and miR-3064-5p expression was upregulated by circGFRA1 knockdown in PC-3 and LNCap cells (Figure 5(c)). The predicted binding sites between circGFRA1 and miR-3064-5p are shown in Figure 5(d), and the dual-luciferase reporter assay results suggested that circGFRA1 can directly bind to miR-3064-5p in PCa cells.

### 3.6. MiR-3064-5p Targets HECTD1

**Figure 6:**
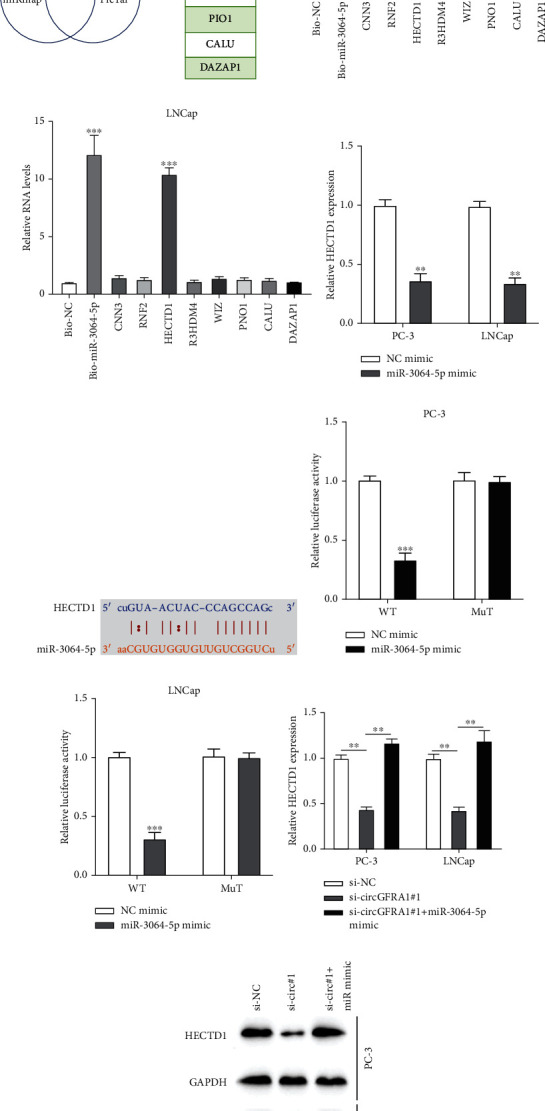
MiR-3064-5p targets HECTD1. (a) Schematic illustration of the predicted mRNA targets of miR-3064-5p. (b and c) Biotinylated RNA pull-down using bio-NC and bio-miR-3064-5p probes to assess the relationship between HECTD1 and miR-3064-5p in PC-3 and LNCap cells using the bio-NC group as control. (d) HECTD1 expression in miR-3064-5p overexpressed cells was detected by qRT-PCR using the NC mimic group as control. (e) Schematic of HECTD1 illustrating the position of the miR-3064-5p binding site. (f and g) Relative luciferase activity in PC-3 and LNCap cells co-transfected with HECTD1-WT or HECTD1-MUT and miR-NC mimic, miR-3064-5p mimic in PC-3, and LNCap cells using the NC mimic group as control. (h and i) HECTD1 expression in si-NC, si-circGRFA1#1, and si-circGRFA1#1+miR-3064-5p mimic transfected PC-3 and LNCap cells was measured by qRT-PCR and western blotting using the si-NC group as control. Data are presented as mean ± standard error. ∗∗*P* < 0.01, ∗∗∗*P* < 0.001.

Of the eight potential mRNA targets of miR-3064-5p (Figure 6(a)), the biotinylated RNA pull-down assay results suggested that HECT domain E3 ubiquitin protein ligase 1 (HECTD1) was the target gene of miR-3064-5p in PCa cells (Figures 6(b) and 6(c)). Also, miR-3064-5p overexpression inhibited HECTD1 expression (Figure 6(d)). The predicted binding sites between miR-3064-5p and HECTD1 are shown in Figure 6(e). The luciferase reporter assay results showed that miR-3064-5p can directly target HECTD1 in PCa cells (Figures 6(f) and 6(g)), and HECTD1 expression was downregulated by circGFRA1 knockdown and upregulated when miR-3064-5p mimic was co-transfected into PCa cells (Figures 6(h) and 6(i)). Taken together, these results suggest that circGFRA1 can regulate HECTD1 expression in PCa cells through sponging miR-3064-5p.

### 3.7. HECTD1 Is Crucial for the Functional Role of circGFRA1 in PCa

**Figure 7:**
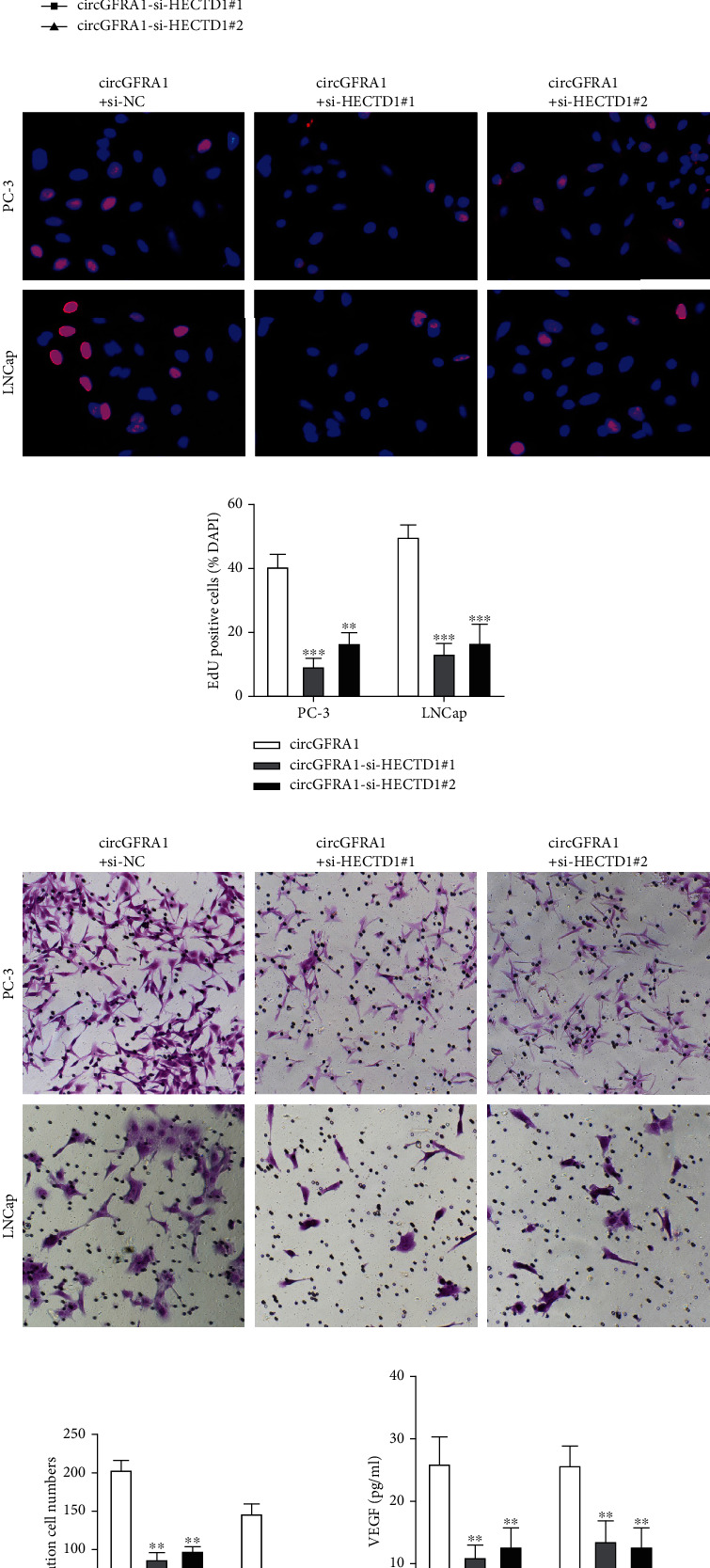
HECTD1 is crucial for the functional role of LMX1B in PCa. (a) HECTD1 expression in vector and circGFRA1 transfected cells measured by qRT-PCR and western blotting using the vector group as control. (b) HECTD1 expression in circGFRA1-si-NC, circGFRA1-si-HECTD1#1, and circGFRA1-si-HECTD1#2 transfected cells measured by qRT-PCR and western blotting. (c) Cell proliferation was detected by CCK-8. (d and e) EdU assay was performed to evaluate cell proliferation. (f and g) Cell migration assessed by the transwell assay. (h) VEGF content of PCa cell supernatants. (i) IL-10 content of PCa cell supernatants. (j) TGF-*β*1 content of PCa cell supernatants. (k and l) The cytotoxicity of CIK cells to PC-3 and LNCap cells was determined via CCK-8 assay using the circGFRA1-si-NC group as control. Data are presented as mean ± standard error. ∗*P* < 0.05, ∗∗*P* < 0.01, ∗∗∗*P* < 0.001.

To determine whether circGFRA1 played a functional role through HECTD1, HECTD1 expression was quantified in circGFRA1 up- or downregulated PCa cells. The mRNA and protein levels of HECTD1 were consistent with the change in circGFRA1 expression (Figures 7(a) and 7(b)) and compared to transfection of circGFRA1 siRNAs alone, and co-transfection with HECTD1 reversed the suppression effect of circGFRA1 knockdown on cell proliferation (Figures 7(c)–7(e)) and migration (Figures 7(f) and 7(g)). Also, HECTD1 overexpression significantly reversed the circGFRA1 overexpression-induced upregulation of VEGF, IL-10, and TGF-*β*1 in the supernatant of PCa cells (Figures 7(h)–7(j)) and rescued the circGFRA1 overexpression-regulated CIK cell-induced cytotoxic activity towards PCa cells (Figures 7(k) and 7(l)), suggesting that HECTD1 is essential for the functional role of circGFRA1 in PCa progression.

## 4. Discussion

PCa is the most common cancer in men and a great threat to the genitourinary health of men [3]. In recent years, great progress has been made in the diagnosis or treatment of PCa with the identification of multiple diagnostic targets such as the main biomarker prostate-specific antigen (PSA), PCa antigen 3 (PCA3), the gene fusion test of TMPRSS2-ERG, circulating tumor cells, lncRNA biomarkers, and microRNA biomarkers [19–22]. PCa therapy involves radical prostatectomy or radical radiotherapy, endocrine therapy [23], and enzalutamide treatment when the PCa progresses to castration-resistant PCa [24]. However, despite the wide application of these new diagnostic biomarkers or treatment strategies, patients remain poor, so novel therapeutic targets for PCa are urgently needed.

There is emerging evidence of the role of circRNA in multiple cancers, including PCa [25, 26]. The present study showed that circGFRA1 was upregulated in PCa tumor tissues compared to normal tissues. CircGFRA1 has been investigated in several cancers, such as nonsmall cell lung cancer, ovarian cancer, breast cancer, and hepatocellular cancer [27–30], but its role in PCa has not been fully elucidated. Our results suggest that circGFRA1 plays an oncogene role in PCa progression, which renewed the profile of circGFRA1 in tumorigenesis progression.

Recently, the interaction between circRNA and transcription factors has been demonstrated to be important for circRNA maintenance and function [31, 32]. For instance, circRNA ARF1 expression in glioma stem cells is regulated by U2AF2 [33]. circRNA circHipk2 expression in C2C12 myoblasts is mediated by Sp1 [34], and circ-FOXP1 in hepatocellular carcinoma cells is regulated by SOX9 [9–11]. Therefore, we investigated the upstream regulator of circGFRA1 in PCa cells showing that circGFRA1 is regulated by LMX1B, which has previously been shown to be involved in many cancers, including ovarian, esophageal, and glioma [35–37].

Furthermore, circGFRA1 upregulated HECTD1 expression to promote PCa progression by sponging miR-3064-5p. Interestingly, despite the investigations of miR-3064-5p or HECTD1 in various cancers [38–42], no study has been conducted in PCa. The present study is the first to explore the biological or mechanical role of miR-3064-5p or HECTD1 in PCa, which might be useful for the basic research conducted in PCa.

Although this study has partially demonstrated the functional role of circGFRA1 in PCa, further investigation of more PCa tissues and the underlying molecular pathway of HECTD1 in PCa cellular behaviors are required to confirm the clinical significance of circGFRA1. In conclusion, the present study has partially revealed the involvement of the LMX1B/circGFRA1/miR-3064-5p/HECTD1 axis in PCa progression, providing new insights for developing novel diagnostic or therapeutic targets for PCa.

## Data Availability

The datasets used and/or analyzed during the current study are available from the corresponding author on reasonable request.
